# Epidemiology of tuberculosis in Mongolia: analysis of surveillance data, 2015–2019

**DOI:** 10.5365/wpsar.2023.14.1.931

**Published:** 2023-03-24

**Authors:** Tsolmon Boldoo, Larissa Otero, Borgil Uranchimeg, Anuzaya Purevdagva, Temuulen Enebish, Oyunchimeg Erdenee, Tauhid Islam, Fukushi Morishita

**Affiliations:** a National Tuberculosis Programme, Ministry of Health, Ulaanbaatar, Mongolia.; b Instituto de Medicina Tropical Alexander von Humboldt, Universidad Peruana Cayetano Heredia, Lima, Peru.; c World Health Organization Regional Office for the Western Pacific, Manila, Philippines.; d World Health Organization Representative Office for Mongolia, Ulaanbaatar, Mongolia.

## Abstract

Mongolia has a high tuberculosis (TB) burden. Data from routine paper-based surveillance were used to describe the epidemiology of TB in Mongolia; the data included testing presumptive TB cases, TB notifications, drug-resistant cases, treatment outcomes and notifications in prisoners. The proportion of the population tested for TB increased between 2015 and 2019. The number and rate per 100 000 population of TB notifications decreased between 2015 and 2018 and then increased in 2019. Most TB notifications in 2019 were in the capital, Ulaanbaatar (59.3%), followed by the central (16.8%), Khangai (10.4%), east (8.5%) and west (5.0%) regions. About half of TB notifications nationally were bacteriologically confirmed (45.4% in 2015, 48.1% in 2019), with the proportion of bacteriologically confirmed TB per province or district varying from 0% to 66%. High TB notification rates were observed in 2019 for males aged 15–54 years (202 per 100 000 population) and females aged 15–34 years (190 per 100 000 population). Treatment success for all forms of TB was 90% in 2019 but was below the 90% target for bacteriologically confirmed cases. Between 2015 and 2019, the number of RR/MDR-TB notifications ranged from 265 to 211. The Mongolian National Tuberculosis Programme needs to continue its efforts in TB control, to further increase the programmatic impact and reduce the TB burden. It is recommended that Mongolia continue to increase TB screening, the use of Xpert testing, contact investigations and preventive treatments, and targeting interventions to the high-burden areas identified in this subnational analysis.

In 2021, there were an estimated 10.6 million cases and 1.4 million deaths from tuberculosis (TB) globally, with 14% of cases in the Western Pacific Region. (*1*) The first national TB prevalence survey in Mongolia was conducted in 2014–2015; it estimated the pulmonary TB prevalence to be 441 per 100 000 population, and the prevalence of all forms of TB to be 757 per  100 000 population. (*2*) Based on the newly available data, TB incidence was re-estimated by the World Health Organization (WHO) to be 437 (uncertainty range: 224–719) per 100 000 population, (*3*) ranking Mongolia among the 30 countries with the highest TB incidence in the world. (*1*)

Mongolia’s National Tuberculosis Programme (NTP) surveillance system is a combination of a paper-based aggregated system and a digital case-based system that covers TB cases from screening through to completion of treatment. Subnational analysis of key TB indicators and trends over time is useful for programmatic decision-making and helps to increase programmatic impact where interventions can be tailored to local dynamics. (*4*, *5*) Through analysis of routine surveillance data, we report TB epidemiology and key programmatic indicators at the national and subnational levels for 2015–2019.

## Methods

### Description of the surveillance system

In Mongolia, TB cases can be detected through passive case detection, in which symptomatic individuals attending primary care facilities are screened for TB. Those who present with a persistent cough are referred to a TB dispensary for a diagnostic evaluation by sputum smear microscopy. If smear-positive, the patient is registered as a confirmed TB case and is started on treatment; if smear-negative, a chest X-ray is conducted. Since 2017, the Xpert MTB/RIF test is also conducted where possible.

Cases can also be detected through screening of close contacts of TB cases or through active case finding in high-risk groups (e.g. people living with HIV, miners and prisoners). Contacts and high-risk groups are tested through symptom screening and chest X-ray, and the tuberculin skin test (TST) is also used for child contacts. Contacts and those from high-risk groups who are positive on screening are referred for diagnostic evaluation to TB dispensaries.

All cases are registered on paper forms at the TB dispensaries; staff then compile aggregate monthly reports of notifications and treatment outcomes and send them to the provincial level, where they are aggregated each quarter and sent to the national level, where they are collated and reviewed for timeliness, completeness and accuracy by an NTP statistician. The system uses standardized TB collection forms updated with the latest WHO reporting framework for TB case detection and treatment outcomes. (*6*) From 2018, the digital case-based system, TUBIS, has been used to collect individual case data, capturing 90% of the data from the paper-based system.

### Data analysis

National TB surveillance data for 2015–2019 were retrospectively analysed, using data sourced from the aggregated paper-based system. Rates were calculated using population projections from the National Statistical Office of Mongolia, and vital and civil registration from the 2010 census for the denominator. Analysis included testing of presumptive TB cases and number of notifications by age, sex, patient type and location, drug-resistant cases, treatment outcomes and notifications in prisoners.

Patient type was classified into bacteriologically confirmed TB, extra-pulmonary TB, clinically diagnosed TB and other previously treated TB. Subnational analysis was conducted for the east, central, Khangai and west regions, plus the capital Ulaanbaatar. Regions were further analysed by their provinces, and Ulaanbaatar by its districts. Drug resistance categories included cases with mono-drug and poly-drug resistance, and cases with rifampicin resistance or multidrug resistance (RR/MDR). Treatment outcomes for bacteriologically confirmed TB cases included treatment success, treatment failure, death and loss to follow-up (LTFU). Stata software (Stata Corp, 16.0, College Station, TX, United States of America) was used for analysis. RR/MDR-TB treatment outcomes were analysed for the 2017 cohort only because in most cases treatment duration is 24 months.

## Results

### Testing of presumptive TB cases

**Fig. 1:**
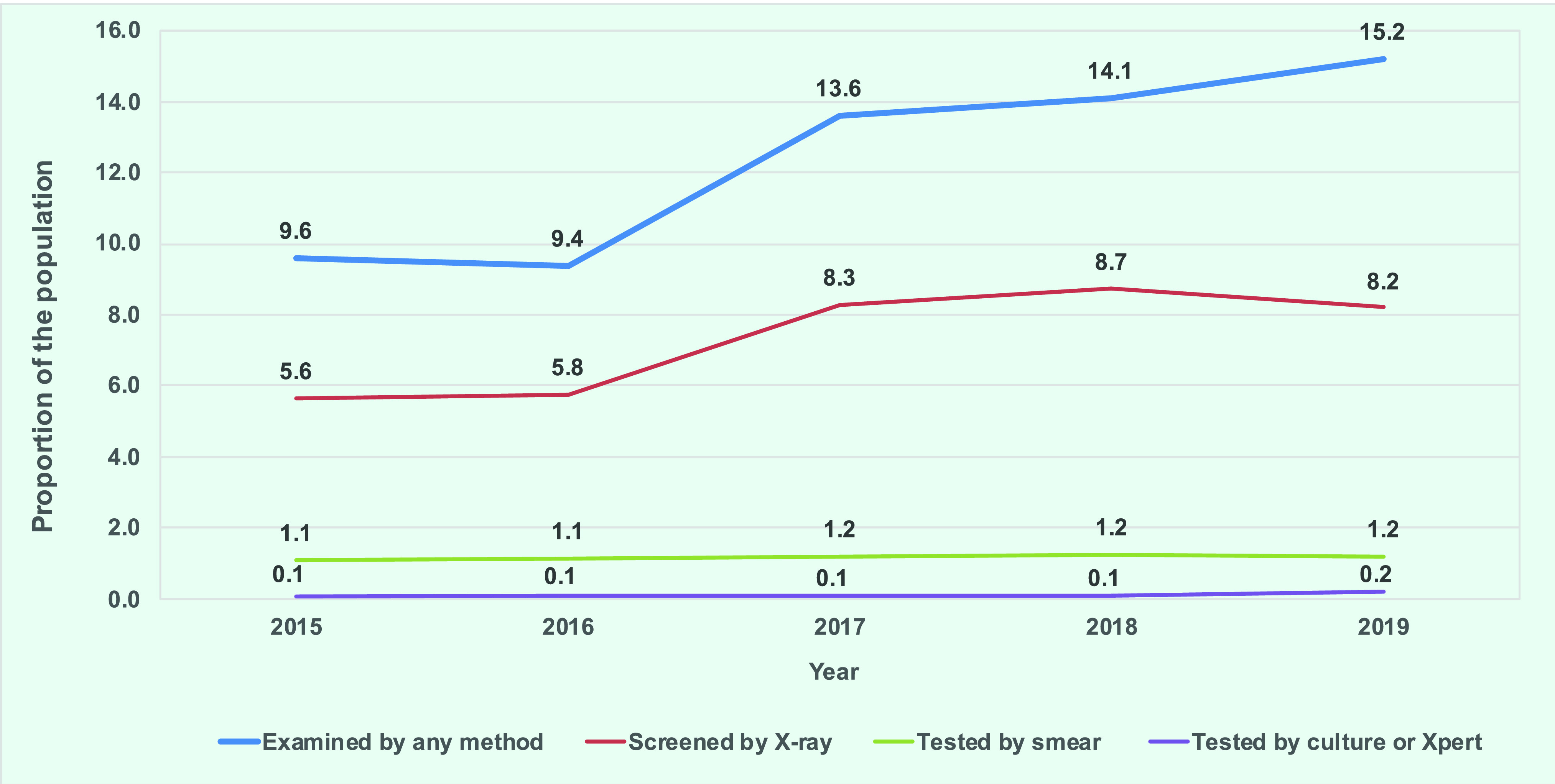
Proportion of the population examined for TB by test, Mongolia, 2015–2019

The proportion of the population tested for TB increased from 2015 to 2019, as did the proportion of the population tested by X-ray (**Fig. 1**).

[**insert** Figure 1]

In 2019, 85.7% (*n* = 4664/5422) of registered TB contacts were tested, with 4.0% (*n* = 185) diagnosed with TB. These were slight increases compared with 2015 (81% and 3.3%, respectively). The proportion of child contacts (aged 0–14 years) who were TST-positive was 20.2% (*n* = 424/2102) in 2019, an increase from 16.7% (*n* = 324/1936) in 2015. The proportion of TST-positive child contacts who started on TB-preventive treatment was 71.2% (*n* = 302/424) in 2019, an increase from 46.0% (*n* = 149/324) in 2015.

The smear positivity rate was 8% (*n* = 2376/28 753) in 2019, a decrease from 12% (*n* = 2812/23 703) in 2015. This varied subnationally, from 5% (*n* = 570/14 301) in 21 provinces to 10% (*n* = 3565/36 714) in Ulaanbaatar. Of the 17 854 Xpert MTB/RIF tests done in 2019, 3070 (17.2%) were MTB-positive, of which 261 (8.5%) were RR-TB.

### Case notifications by patient type

**Fig. 2:**
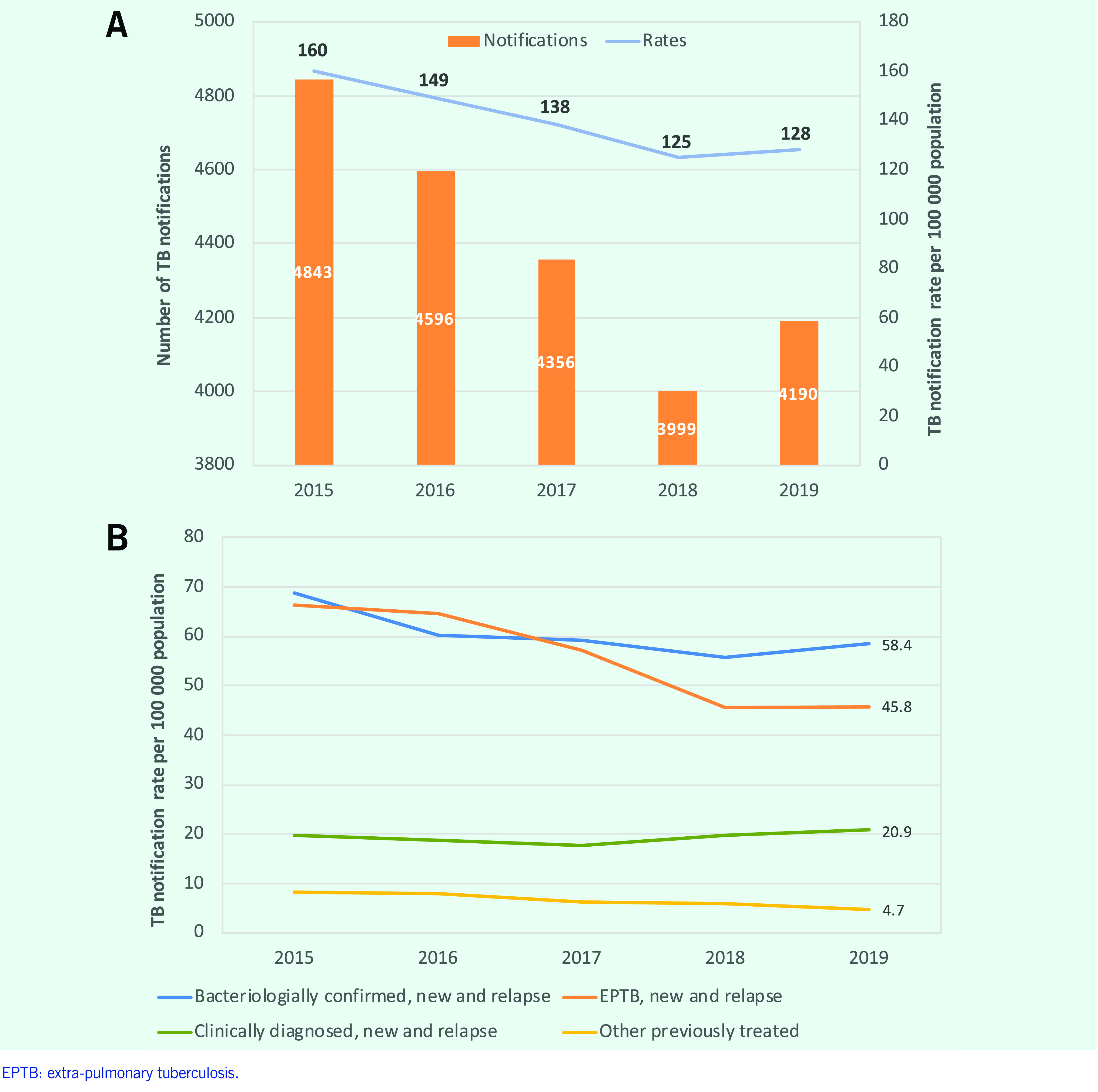
(A) Number and rate per 100 000 population of TB notifications, and (B) TB notification rates per 100 000 population by type of TB, Mongolia, 2015–2019

The number and rate of notified TB cases decreased between 2015 and 2018, and then increased in 2019  (**Fig. 2A**). The increase in 2019 was observed for bacteriologically confirmed TB, extra-pulmonary TB and clinically diagnosed TB (**Fig. 2B**).

In 2019, there were 133 per 100 000 population new and relapse TB cases notified, representing 31% of the WHO-estimated incident cases (*n* = 14 000). (*7*) Of all TB notifications in 2019, 85.4% (*n* = 3624) were new cases, 11.0% (*n* = 465) were relapse cases, 3.4%(*n* = 146) were cases requiring retreatment after treatment failure or LTFU and 0.2% (*n* = 9) had unknown TB treatment history.

[**insert** Figure 2]

The combined proportion of cases requiring retreatment and relapse cases increased from 13.2%  (*n* = 652/4935) in 2015 to 14.4% (*n* = 611/4244) in 2019. The proportion of extra-pulmonary TB cases decreased from 41.9% (*n* = 2068/4935) in 2015 to 35.7% (*n* = 1513/4244) in 2019. Bacteriologically confirmed TB cases comprised about half of all TB cases (45.5%  [*n* = 2244/4935] in 2015 and 48.1% [*n* = 2041/49244] in 2019). Of the pulmonary cases, 74.8%  (*n* = 2043/2731) were bacteriologically confirmed in 2019.

### Subnational case notifications

**Fig. 3:**
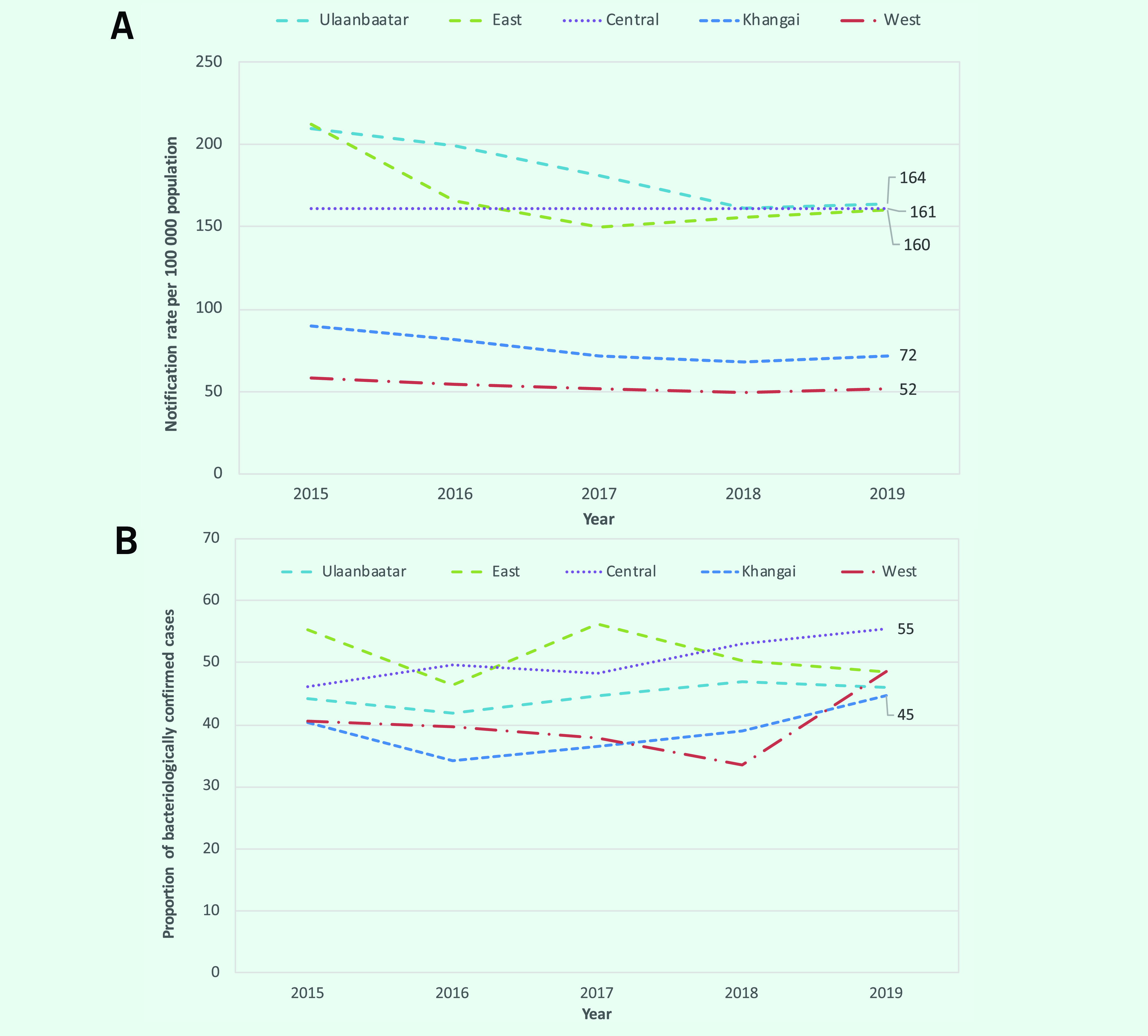
(A) TB notification rates per 100 000 population by region, and (B) proportion of TB cases that were bacteriologically confirmed by region, Mongolia, 2015–2019

**Fig. 4:**
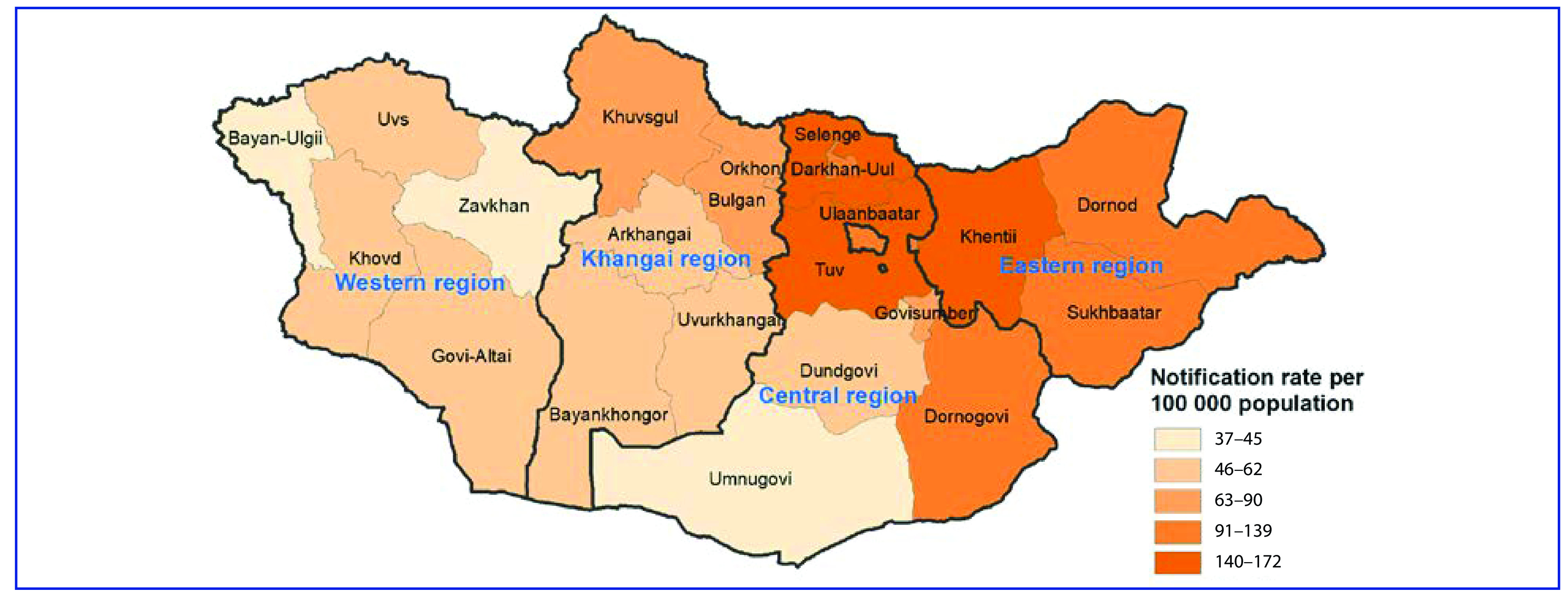
Map of TB notification rates per 100 000 population by province, Mongolia, 2019

**Fig. 5:**
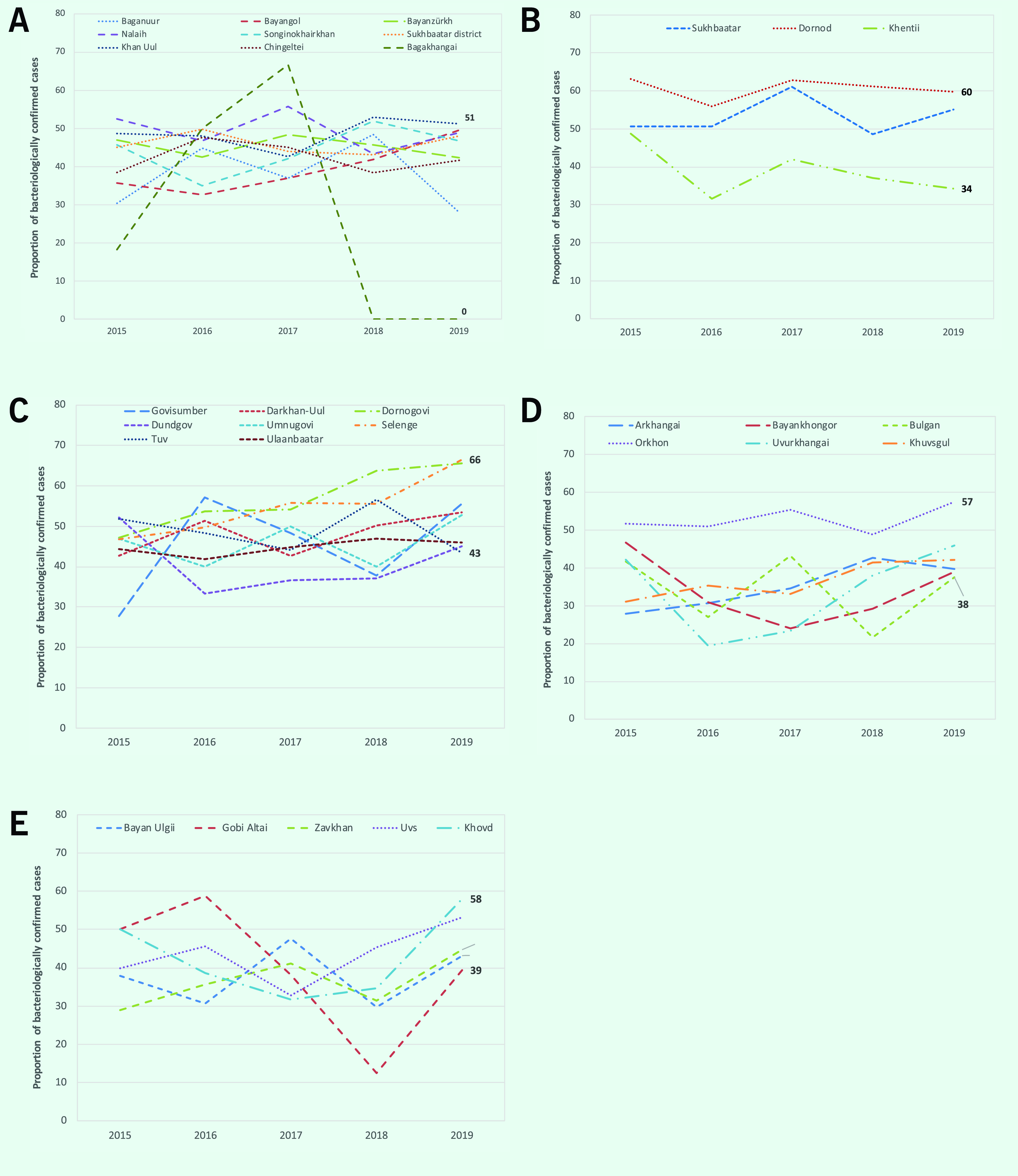
Proportion of TB cases that were bacteriologically confirmed by district in Ulaanbaatar (A) and by province in the east (B), central (C), Khangai (D) and west (E) regions, Mongolia, 2015–2019

Most notifications in 2019 occurred in Ulaanbaatar, followed by the central (excluding Ulaanbaatar), Khangai, east and west regions (**Table 1**). The notification rate increased in the east region from 2017, and in the other four regions from 2018 (**Fig. 3A**). The proportion of cases that were bacteriologically confirmed increased from 2016 in Ulaanbaatar and Khangai, from 2017 in central and from 2018 in the west region (**Fig. 3B**). Notification rates per 100 000 varied substantially across provinces in 2019, ranging from 37 to 172 (**Fig. 4**). In 2019, the proportion of bacteriologically confirmed TB per province or district within each region varied substantially (Ulaanbaatar: 0–51%, east: 34–60%, central: 43–66%, Khangai: 38–57% and west: 39–58%) (**Fig. 5**).

[**insert** Figure 3]

[**insert** Figure 4]

[**insert** Figure 5]

**Table 1:** Number and rate per 100 000 population of TB notifications by treatment history and proportion of retreatment and extra-pulmonary TB by province or district, Mongolia, 2019

Region and provinces or districts	Population	Notifications (N)		Rates per 100 000 population		Percentage (%)	
		New and relapse	Previously treated	New and relapse	Previously treated	Retreatment	Extra- pulmonary TB
**Ulaanbaatar**	**1 515 593**	**2359**	**127**	**156**	**8**	**5.1**	**37.1**
Baganuur	28 570	25	0	88	0	0	28.0
Bayangol	225 840	318	7	141	3	2.2	48.0
Bayanzurkh	361 689	638	52	176	14	7.5	48.0
Nalaih	37 659	40	5	106	13	11.1	37.8
Songinokhairkhan	327 580	581	30	177	9	4.9	38.3
Sukhbaatar	144 409	154	0	107	0	0	35.7
Khan-Uul	187 278	246	10	131	5	3.9	25.8
Chingeltei	148 977	304	16	204	11	5.0	38.4
Bagakhangai	4123	0	0	0	0	0	0
**East**	**221 764**	**348**	**7**	**157**	**3**	**2**	**30.7**
Khentii	77 493	139	1	179	1	0.7	40.0
Dornod	81 519	112	5	137	6	4.3	20.5
Sukhbaatar	62 752	97	1	155	2	1.0	29.6
**Central**	**515 025**	**696**	**7**	**135**	**1**	**1**	**31.0**
Selenge	110 757	174	2	157	2	1.1	25.0
Umnugovi	67 955	36	0	53	0	0	44.4
Tuv	94 956	173	2	182	2	1.1	36.6
Darkhan-Uul	106 470	172	2	162	2	1.1	29.3
Govisumber	17 862	18	0	101	0	0	27.8
Dundgov	46 866	31	0	66	0	0	51.6
Dornogovi	70 159	92	1	131	1	1.1	23.7
**Khangai**	**604 784**	**427**	**7**	**71**	**1**	**2**	**50.5**
Orkhon	106 810	90	4	84	4	4.3	52.2
Uvurkhangai	116 922	61	0	52	0	0	58.8
Bayankhongor	88 514	59	0	67	0	0	51.3
Arkhangai	95 857	58	0	61	0	0	58.0
Khuvsgul	134 530	111	3	83	2	2.6	44.7
Bulgan	62 151	48	0	77	0	0	40.0
**West**	**410 507**	**209**	**3**	**51**	**1**	**1**	**39.2**
Uvs	83 766	46	1	55	1	2.1	23.4
Bayan-Ulgii	106 810	44	0	41	0	0	52.3
Zavkhan	72 801	36	2	49	3	5.3	52.6
Khovd	89 021	50	0	56	0	0	36.0
Gobi-Altai	58 109	33	0	57	0	0	33.3

### Case notifications by sex and age in 2019

**Fig. 6:**
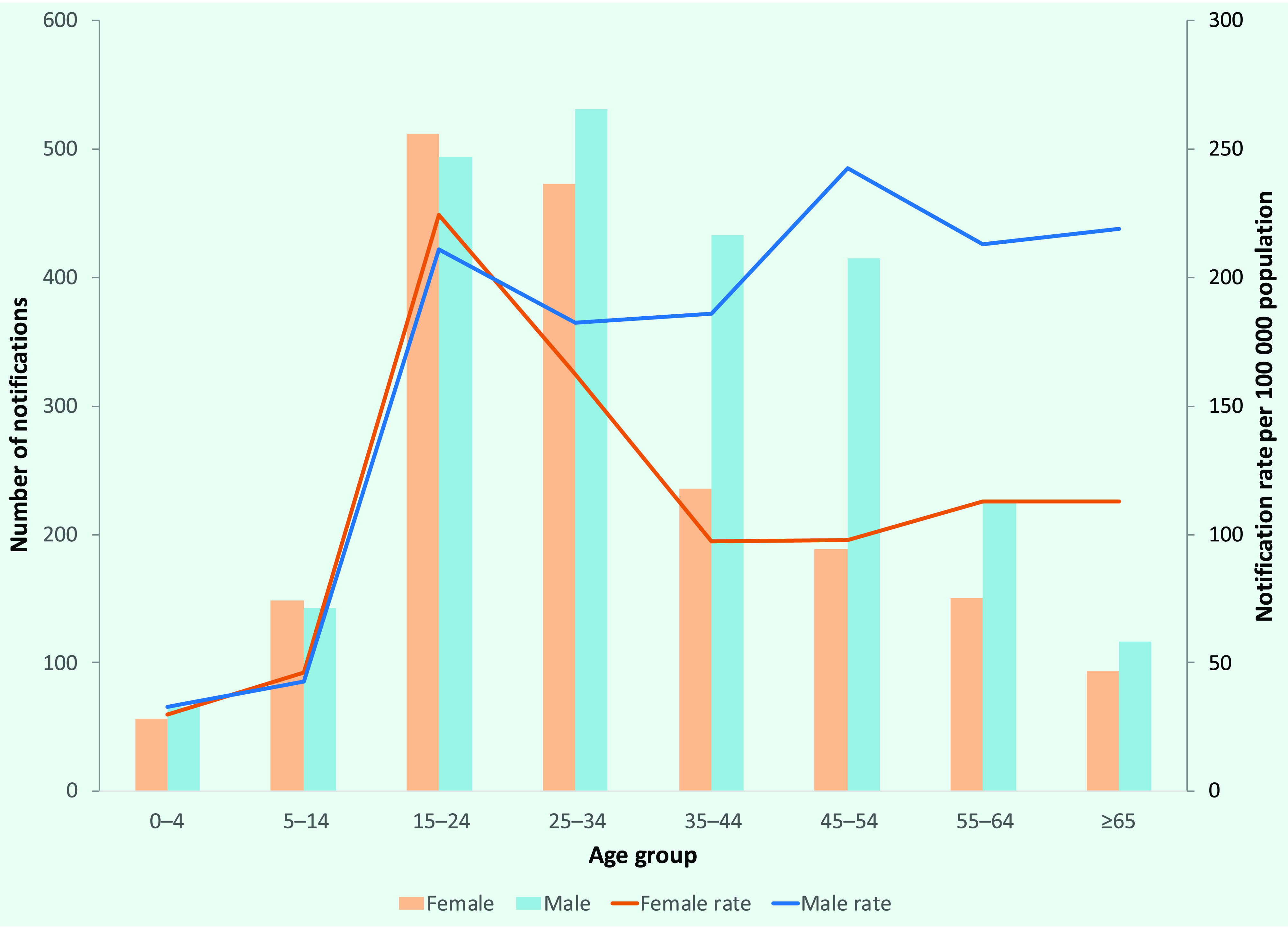
Number and rate per 100 000 population of TB notifications by sex and age group, Mongolia, 2019

**Fig. 7:**
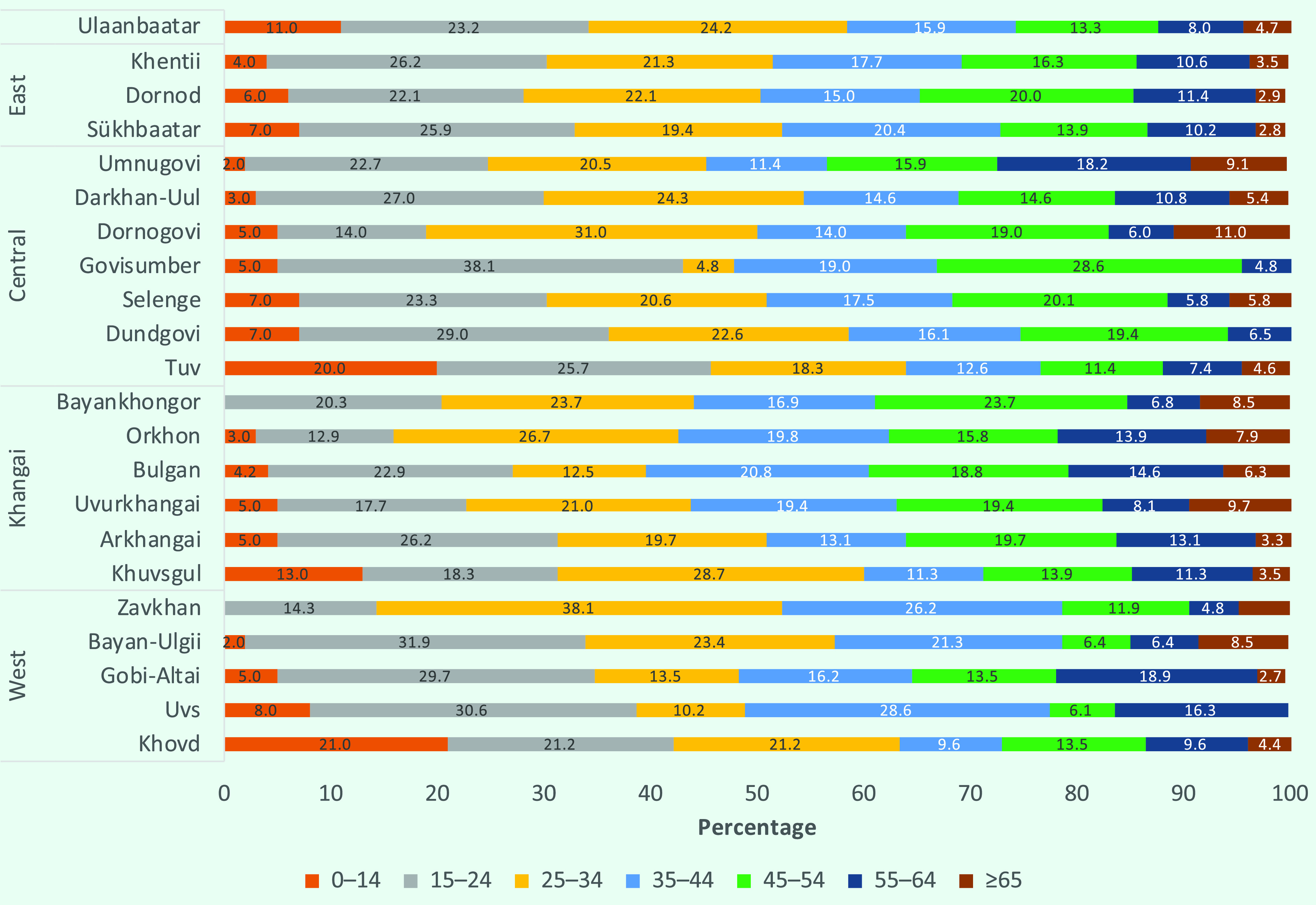
Proportion of TB notifications by age group and province, Mongolia, 2019

The highest numbers of case notifications in 2019 were seen in males aged 15–54 years and females aged 15–34 years (**Fig. 6**). This distribution varied by TB type: 57.2% of new cases were male, with a mean (± standard deviation [SD]) age of 33 (± 17.3) years, whereas 66.9% of relapse cases were male, with a mean age of 40 (± 13.9) years. In 2019, 9.1% (*n* = 415) of TB notifications were aged under 15 years and 2.7% (*n* = 121) were aged under 5 years. Subnational analysis showed large variations in the proportion of cases by age group and among children, with some provinces having no paediatric TB notifications or only small numbers of such notifications (**Fig. 7**).

[**insert** Figure 6]

[**insert** Figure 7]

### Drug-resistant TB

**Fig. 8:**
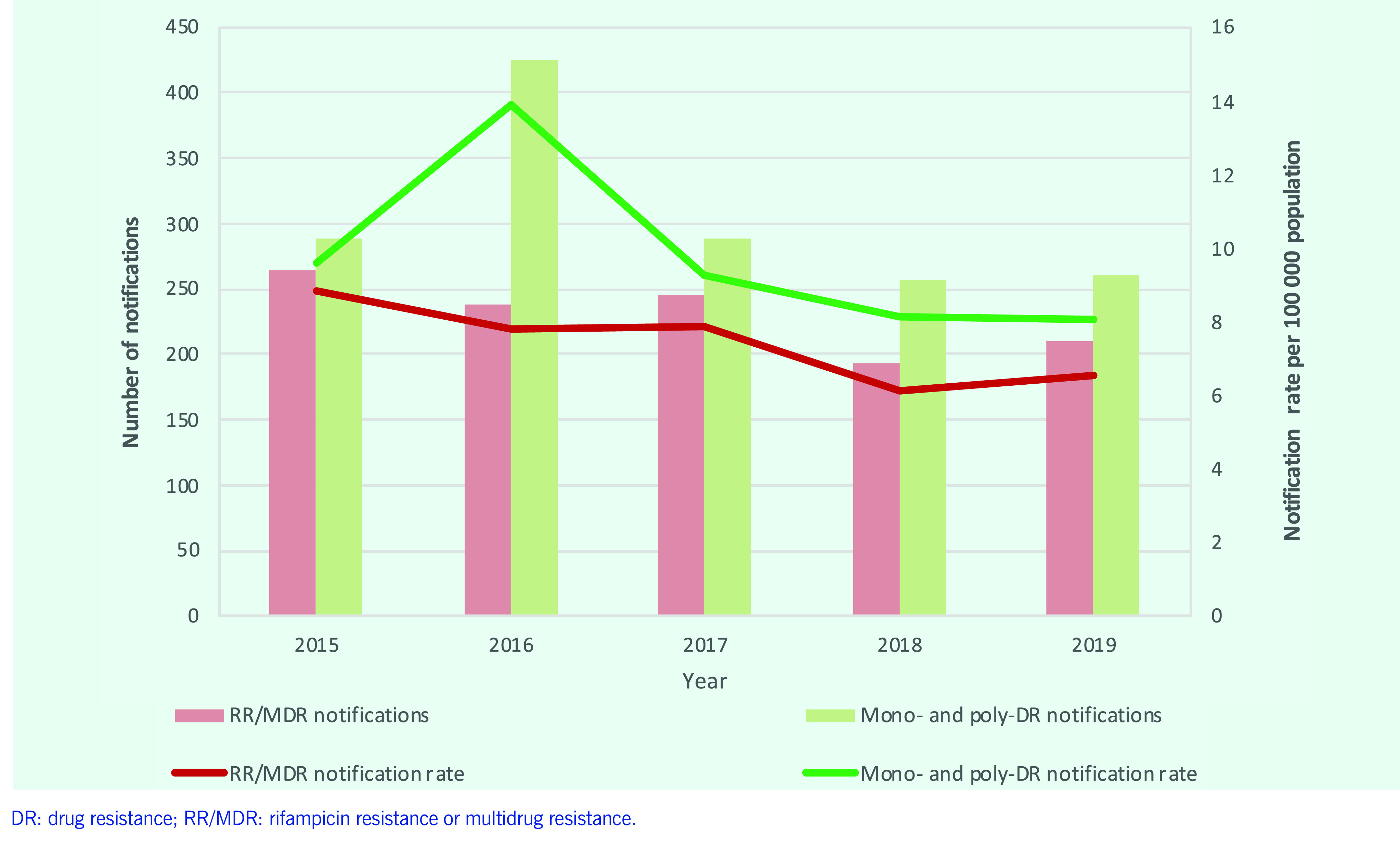
Number of TB notifications and notification rate per 100 000 population by drug-resistant TB categories, Mongolia, 2015–2019

Between 2015 and 2019, the number of RR/MDR-TB notifications ranged from 265 to 211 (**Fig. 8**). In 2019, 211 RR/MDR-TB cases were diagnosed; of these, 92% (*n* = 193) were enrolled in second-line TB treatment, an increase from 85% of the 265 cases in 2015. Seven extensively drug-resistant TB (XDR-TB) cases were diagnosed in 2019.

Of the 211 RR/MDR-TB cases, 46.9% (*n* = 99) were new cases and 41.7% (*n* = 88) were relapse and other previously treated cases. Of new RR/MDR-TB cases, 40.4% were female, similar to the proportion seen in all TB notifications. The mean age for new RR/MDR-TB cases was 34.9 (± 18.4) years, similar to the proportion seen in all TB notifications (33 ± 17.3). The mean age for relapse and other previously treated RR/MDR cases was 41 (± 14.3).

[**insert** Figure 8]

### Treatment outcomes

The proportion of TB notifications with treatment success increased from 88.8% in 2015 to 90.0% in 2019. The proportion of deaths decreased from 4.6% in 2015 to 2.5% in 2019. The proportion of cases that were LTFU was stable (4.6% in 2018), as was the proportion of those not evaluated (0.4% in 2019). Since 2016, the treatment success rate has been above 90% for all types of TB except for bacteriologically confirmed TB cases (85.4%). The death rate was highest among relapse cases (4.8%).

**Fig. 9:**
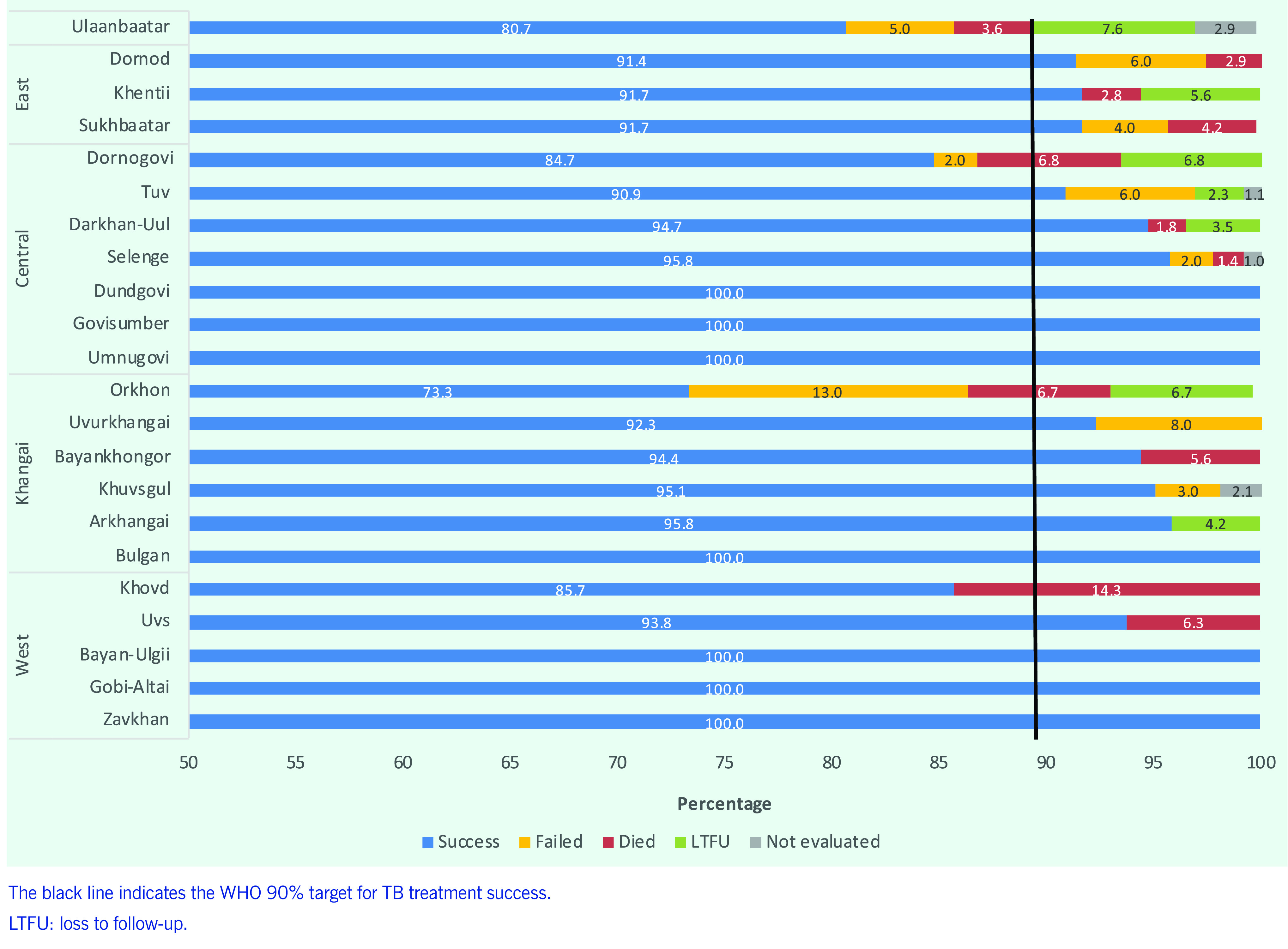
Proportion of bacteriologically confirmed TB notifications by treatment outcomes and province, Mongolia, 2019

In 2019, the treatment success rate for bacteriologically confirmed cases was less than 90% in three provinces (Dornogovi, Khovd and Orkhon) and Ulaanbaatar (**Fig. 9**). Ulaanbaatar reported relatively poor treatment outcomes compared with other provinces; 8% of bacteriologically confirmed cases were LTFU, 5% failed and 4% died.

[**insert** Figure 9]

In 2017, 56% (*n* = 122/216) of RR/MDR-TB patients enrolled in treatment were successfully treated, a slight decrease from 60% in 2015–2016. The LTFU rate among RR/MDR-TB cases increased from 16% in 2016 to 26% in 2017. Of seven XDR-TB cases in the 2017 patient cohort, two (28.6%) were successfully treated, one (14.3%) failed, three (42.9%) died and one (14.3%) was LTFU.

### TB in prisoners

TB notifications in prisoners decreased from 92 (1.9%) in 2015 to 54 (1.3%) in 2019. A higher proportion of bacteriologically confirmed cases (64.3%) and relapse cases (25.9%) were notified than from the national data (58.5% and 11%, respectively).

## Discussion

The results of this TB surveillance analysis demonstrate the progress of the NTP in Mongolia, with increases in the proportion of the population screened for TB, bacteriological confirmation, treatment success and TB-preventive treatment in children. Intensification of case-finding activities through the expansion of Xpert testing and sustaining treatment success, particularly in Ulaanbaatar, will probably increase the impact of the NTP and reduce the national TB burden.

The number and rate of TB notifications decreased in 2015–2018 and increased in 2019, despite increases in screening. Similar trends have also been observed in other high-burden countries such as Cambodia, where estimated TB incidence is declining. (*4*, *5*) The expansion of X-ray and Xpert testing and the strengthening of the specimen transportation system may have resulted in an increase in notifications and an increased proportion of bacteriological confirmation in 2019. However, WHO estimates that the TB notification system is detecting only 31% of TB cases in the country. (*1*) To fill this gap, the NTP needs to intensify its efforts in screening high-risk populations. (*7*)

The WHO-estimated incidence of RR/MDR-TB for Mongolia was one of the highest among countries in the Western Pacific Region. (*1*) However, the number of RR/MDR-TB notifications did not increase during the study period, highlighting a case-detection gap that is also found in other countries. (*4*) To respond to the burden of DR-TB, there is an urgent need to increase the coverage of Xpert as an initial diagnostic test and reduce diagnostic delays.

The high caseload in younger age groups suggests recent transmission, emphasizing the need to expand and accelerate case detection. Exposure to tobacco and solid fuels for heating has been significantly associated with bacteriological TB, (*8*) which may contribute to the higher rates in these age groups. The proportion of TB in children varied widely across provinces. As in many settings globally, there is a need to strengthen the capacities of physicians in diagnosing paediatric TB and expand Xpert testing in children to increase correct and timely diagnoses. (*1*, *9*) TB among prisoners decreased during the study period, as it did in the previous decade. (*10*) Furthermore, the proportion of relapse cases among prisoners was more than double that of the general population.

A decrease in the proportion of deaths of TB cases has improved overall treatment outcomes, but because of persistently high rates of LTFU, bacteriologically confirmed TB treatment success rates remain below 90%. Addressing physical barriers to TB services for mobile populations (including nomads) and reducing financial barriers may improve health access for vulnerable patients. The low treatment success rate among DR-TB cases needs attention, especially considering the increase in the notification rate of MDR-TB found in the national drug resistance survey in 2017, compared with that in 2007. (*11*) The use of Xpert as a front-line test and the implementation of a shorter all-oral regimen for MDR-TB treatment should be prioritized. (*12*)

Our analysis is limited to TB cases diagnosed and reported to the NTP; thus, it does not represent all estimated cases of TB in Mongolia. The 70% case-detection gap estimated at the national level (*1*) is likely to vary between provinces and this was not detected by our analysis. A full transition to the digital case-based system and discontinuing the paper-based system would bolster routine data analysis because individual case data provide more detail than the aggregate data.

The Mongolian NTP needs to continue its efforts in TB control to achieve further progress. Expanding and accelerating case detection with Xpert and ensuring the treatment success of bacteriologically confirmed TB would probably reduce the TB burden. Other priorities are addressing transmission in men and young adults, and strengthening paediatric TB diagnosis. The focus should be on Ulaanbaatar because it has higher notification rates and suboptimal treatment outcomes, and overcrowding and pollution that increase the risk of transmission. Advancing a multisectoral response is critical to addressing social determinants of TB such as indoor air pollution.

TB surveillance data provide an opportunity to conduct subnational analyses, to inform districts of their comparative epidemiological trends and programmatic performance. National and subnational TB programmes can tailor and target interventions addressing local-level issues identified in routine analysis, contributing to ending TB by 2030.
